# Maximum field emission current density of CuO nanowires: theoretical study using a defect-related semiconductor field emission model and *in situ* measurements

**DOI:** 10.1038/s41598-018-20575-y

**Published:** 2018-02-01

**Authors:** Zufang Lin, Peng Zhao, Peng Ye, Yicong Chen, Haibo Gan, Juncong She, Shaozhi Deng, Ningsheng Xu, Jun Chen

**Affiliations:** State Key Laboratory of Optoelectronic Materials and Technologies, Guangdong Province Key Laboratory of Display Material and Technology, School of Electronics and Information Technology, Sun Yat-sen University, Guangzhou, 510275 China

## Abstract

In this study, we proposed a theoretical model for one-dimensional semiconductor nanowires (NWs), taking account of the defect-related electrical transport process. The maximum emission current density was calculated by considering the influence of Joule heating, using a one-dimensional heat equation. The field emission properties of individual CuO NWs with different electrical properties were studied using an *in situ* experimental technique. The experimental results for maximum emission current density agreed well with the theoretical predictions and suggested that multiple conduction mechanisms were active. These may be induced by the concentration of defects in the CuO NW. The concentration of defects and the transport mechanisms were found to be key factors influencing the maximum field emission current density of the semiconductor NW. As is limited by the change of resistivity with temperature, only thermal runaway can trigger breakdown in CuO NWs.

## Introduction

The field emission properties of one-dimensional semiconductor nanostructures have been extensively investigated, given their potential application as the electron source in field emission devices such as electron microscopes^1^, vacuum electronic devices^2,3^, microwave tubes^4^, X-ray sources^5,6^, and flat panel displays^7–9^. Because of their superior antioxidant properties and physical stability, metal oxide semiconductor nanowires (NWs) have attracted increasing attention, and significant efforts have been made to improve their field emission properties^10–13^. Applications such as microwave vacuum electronic devices, X-ray tubes, and high-power terahertz sources require field emitters with high emission current density^14,15^. However, this is challenging to achieve for the majority of metal oxide semiconductor NWs^16,17^. To allow these materials to find a wider range of applications, therefore, further investigation of their emission mechanisms is needed.

In the course of field emission, the temperature of the emitter increases as Joule heating is induced by the emission current and the resistance of the emitter. This increase in temperature may be balanced by thermal conduction through the substrate and radiation to the atmosphere. If thermal equilibrium can be achieved, emission will stabilize. However, if the current is increased sufficiently, thermal equilibrium will be broken in a process referred to as thermal runaway, or the temperature will increase continuously until it exceed the melting point of the emitter or substrate. Under either of these conditions, a breakdown is inevitable, placing limits on the maximum current that can be obtained from a field emitter. While thermal conductivity and geometric parameters play important roles, the resistance of the emitter is also a key parameter, which is influenced by the electron transport process. In the case of a semiconducting NW field emitter, with its small size effect and vulnerability to defects, the temperature-dependent electron transport mechanism becomes more complex. The precise mechanisms by which the transportation process affects the resistance of the emitter and thus determines the maximum emission current have yet to be clarified. More complete knowledge is required to allow the design and fabrication of semiconductor NW field emitters capable of achieving a high emission current.

Significant efforts have been made to investigate the relationship between the electrical properties and field emission properties of one-dimensional field emitters^18–23^. Using a quasi-dynamic method, Huang *et al*.^24^ proposed and investigated a physical mechanism for initiating the breakdown of carbon nanotubes under vacuum. The critical field, critical emission current density, and critical temperature were derived. However, as CNT is a metal-like material, the study adopted a classical field emission model, and the relationship between *R* and *T* was described using a general formula. She *et al*.^25^ found a strong correlation between the turn-on field of a one-dimensional (1D) ZnO nanostructure and the resistance of the emitters. Nanostructures with lower resistance also had lower turn-on and threshold fields. Shao *et al*.^26^ reported that the low field emission current density of CuO NWs was due to their high resistance. However, no detailed experimental investigations or theoretical discussions of the transport processes that influence the field emission of semiconductor NWs have yet been reported. In most semiconductor materials, resistance decreases as the temperature increases, which is different from the behavior of metals. The dynamic process by which thermal equilibrium is reached will be untypical, and the effect of this on the achievement of a higher maximum current density remains unclear. Further investigation of the role played by the transport process in limiting the maximum current density that can be achieved by a semiconductor NW field emitter is needed.

This study proposed a theoretical field emission model for 1D semiconductor NWs, taking into account the contributions made by the concentration of defects and the electrical transport mechanism. An iterative algorithm was used to simulate the dynamic field emission process. The study investigated the roles played by defects concentration and by the electrical transport mechanism, and our theoretical model demonstrated that both are important in determining the maximum field emission current density that can be achieved by a CuO NW. The experimentally measured maximum emission current density agreed well with the theoretical results, once the possibility of multiple conduction mechanisms in the NWs was assumed. Due to the confinement of the conduction mechanism, thermal runaway is the only process that can lead to breakdown in a CuO NW.

## Results

### Field emission model

**Figure 1:**
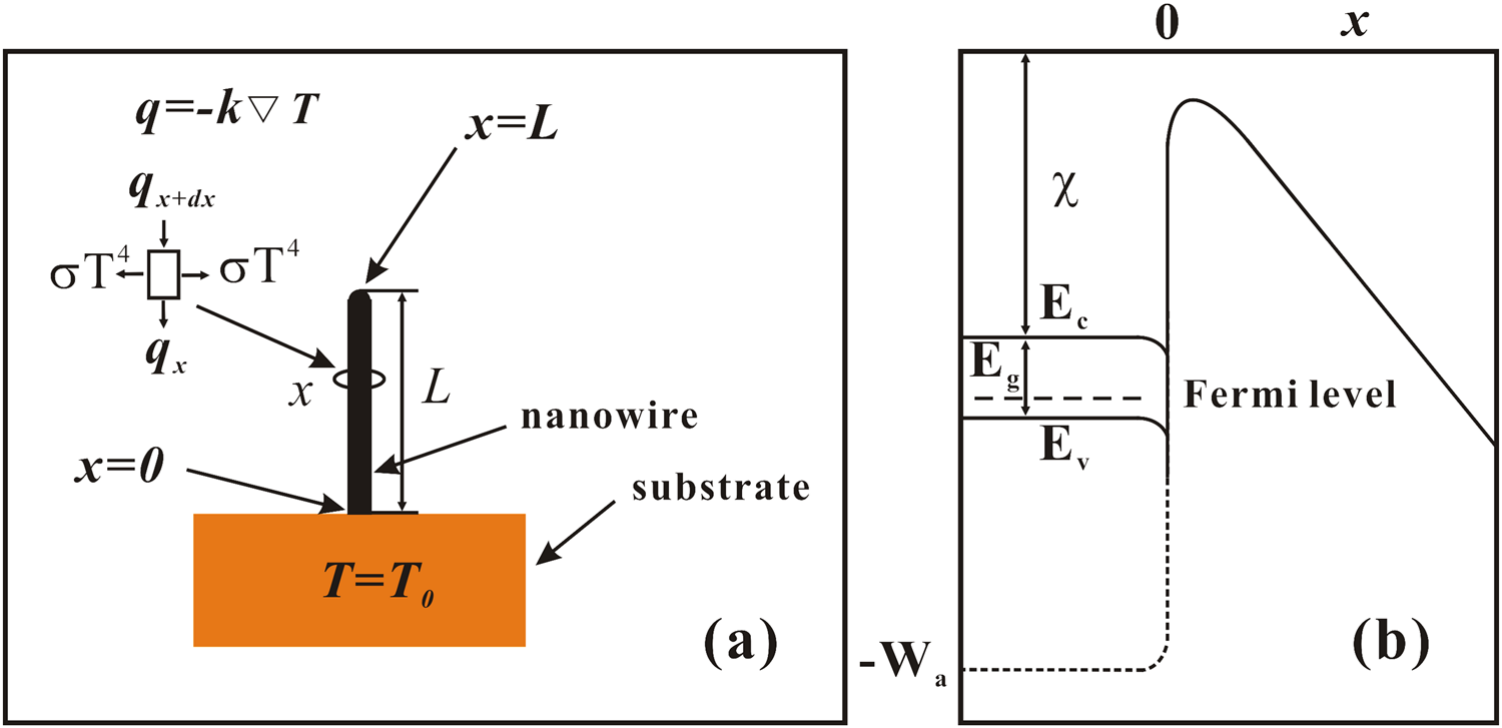
(**a**) Field emission model of the CuO NW. (**b**) Diagram of the electron potential energy of a semiconductor surface.

Figure 1(a) shows the CuO NW assumed by our field emission model. The contact point between the NW and the substrate was set as the zero point (*x* = 0), and the length of the NW was denoted as *L*. When considering the thermal effect, the temperature-dependent heat equation was expressed following^24^:

1 $${I}^{2}R({T}_{x}){L}^{-1}dx+\pi {r}^{2}k\frac{{\partial }^{2}{T}_{x}}{\partial {x}^{2}}dx-2\pi rdx\sigma ({T}_{x}^{4}-{T}_{0}^{4})=0.$$

Here, *I* is the field emission current, *R*(*T*_*x*_) is the resistance of the NW, *T*_*x*_ is the temperature at the *x* point, *r* is the radius of the NW, *k* is the heat conduction coefficient, and σ = 1 is the Stefan–Boltzmann constant. The boundary condition of Eq. (1) was derived following^27^:

2 $$\frac{\partial {T}_{A}}{\partial x}=\frac{-\sigma ({T}_{A}^{4}-{T}_{0}^{4})-j{E}_{N}}{k}.$$

Here, *T*_*A*_ is the temperature at the apex of the NW, *T*_0_ is both the temperature of the substrate and the initial temperature, *j* is the field emission current density, *E*_*N*_ = −*πkT*_*A*_cot(*πT*_*A*_/2*T*_*i*_), and ${T}_{i}=\frac{5.32\times {10}^{-5}F}{{\varphi }^{1/2}}$.

By assuming that the local electric field is *F*, the emitted current density can be expressed as follows^24,28,29^:

3 $$\begin{array}{rcl}j(E,{T}_{A}) & = & \frac{4\pi me{k}_{B}{T}_{A}}{{h}^{3}}\{{\int }_{-{W}_{a}}^{-{E}_{v}}{[1+{e}^{\frac{8\pi {(2m{|w|}^{3})}^{\frac{1}{2}}}{3heF}\sigma (Y)}]}^{-1}\times \,\mathrm{ln}(1+{e}^{\frac{-w+\xi }{{k}_{B}{T}_{A}}})dw\\  &  & +\,{\int }_{-{E}_{c}}^{-{w}_{l}}{[1+{e}^{\frac{8\pi {(2m{|w|}^{3})}^{\frac{1}{2}}}{3heF}\sigma (Y)}]}^{-1}\times \,\mathrm{ln}(1+{e}^{\frac{-w+\xi }{{k}_{B}{T}_{A}}})dw\}\\  &  & +\,\frac{4\pi me{k}_{B}{T}_{A}}{{h}^{3}}{\int }_{-{w}_{l}}^{\infty }\mathrm{ln}(1+{e}^{\frac{-w+\xi }{{k}_{B}{T}_{A}}})dw\end{array}$$

Here, *m* is the electron mass, *k*_*B*_ is the Boltzmann constant, *h* is Planck’s constant, *e* is the elementary charge, *E*_*c*_ is the conduction band energy, *E*_*v*_ is the valance band energy, *ξ* is the Fermi level, $\xi ={E}_{v}-{k}_{B}T\,\mathrm{ln}(\frac{{N}_{A}}{{N}_{V}})$, ${N}_{V}=2\frac{{(2\pi m{k}_{B}T)}^{3/2}}{{h}^{3}}$^30^, *N*_*A*_ is the defect concentration, *σ*(*Y*) is the elliptic function ${w}_{l}=-{[{e}^{3}F/(8\pi \varepsilon )]}^{\frac{1}{2}}$, $Y={[{e}^{3}F/(4\pi \varepsilon {w}^{2})]}^{\frac{1}{2}}$, and *ε* is the dielectric constant of vacuum.

From the Arrhenius plot, the temperature-dependent resistance of the NW could be expressed as follows:

4 $$R({T}_{x})={R}_{0}\,\exp \,(\frac{Q}{{k}_{B}{T}_{x}})={\rho }_{0}\frac{L}{\pi {r}^{2}}\,\exp \,(\frac{Q}{{k}_{B}{T}_{x}}),$$

where *ρ*_0_ is the preexponential factor and the value *R*_0_ (or *ρ*_0_) reflects the concentration of defects in the NW. If the transport mechanism is simplex, *ρ*_0_ will decrease as the concentration of defects increases, and if all defects are ionized, then *ρ*_0_ ∝ 1/*N*_*A*_^30^. In the rest of this paper, therefore, *ρ*_0_ is named the concentration factor. *Q* is a parameter that reflects the transport mechanism^31^, which is influenced in turn by the concentration of defects. In the nearest–neighbour hopping (NNH) mechanism, *Q* will take a low value, typically of a few dozen meV^32^. In the case of thermal activation, *Q* is related to the defect energy level, which is of the order of a hundred meV^33^.

Substituting (4) into (1) and simplifying the function yield

5 $${J}^{2}{\rho }_{0}\,\exp \,(\frac{Q}{{k}_{B}{T}_{x}})dx+k\frac{{\partial }^{2}{T}_{x}}{\partial {x}^{2}}dx-{r}^{-1}dx\sigma ({T}_{x}^{4}-{T}_{0}^{4})=0.$$

## Theoretical calculation

**Figure 2:**
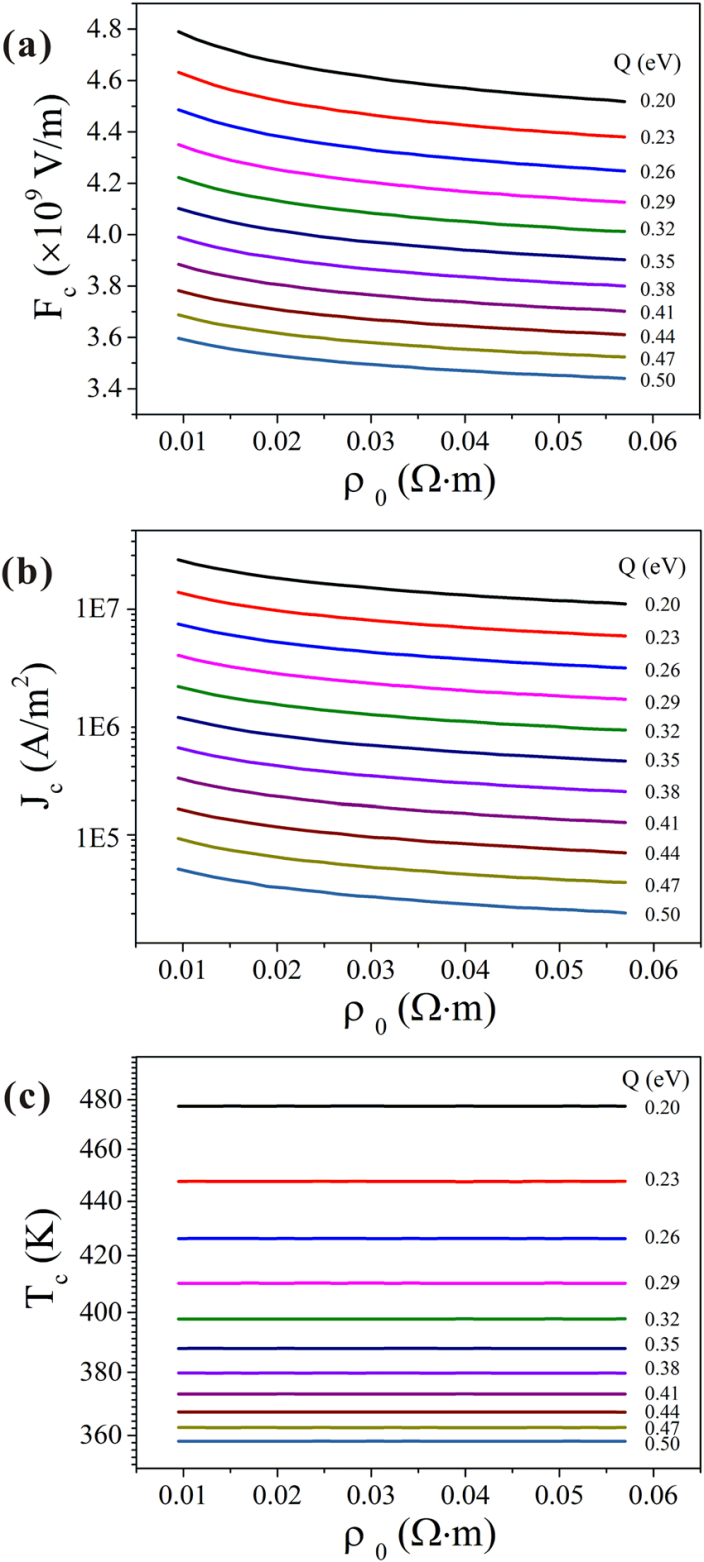
*ρ*_0_ dependence of the maximum local electric field (**a**), maximum emission current density (**b**), and critical temperature *T*_*c*_ (**c**), at different values of *Q*.

This model was applied to calculate the critical temperature at the apex of the NW *T*_*c*_, the maximum emission current *J*_*c*_, and the maximum local electric field *F*_*c*_ of NWs with different defect concentrations and *Q* values. Other parameters used in the calculation were as follows: *E*_*c*_ = −4.07*eV*, *E*_*v*_ = −5.42*eV*^34^, *k* = 33W/(m·K)^35^, *r* = 30 nm, and *L* = 5 μm. An iterative process was used, following^24^. The output is shown in Fig. 2. It is worthy to point out that the field emission area that we used here is *πr*^2^. It is known that the effective emission area is related with the distribution of electric field at the apex of emitter^36,37^. However, the effective emission area will only influence the exact value of the field emission current density. It will have minor effect on our results because we focus on the overall current capability of the nanowire and distribution of current in the nanowire is neglected.

As can be seen, *J*_*c*_ and *F*_*c*_ decreased nonlinearly with respect to *ρ*_0_ of the NWs and therefore increased nonlinearly as the concentration of defects increased. Further, a significant decrease in *J*_*c*_ and *F*_*c*_ was observed as *Q* increased. The value of *T*_*c*_ depends mainly on that of *Q* (Supplementary Fig. S1 shows an example of the relationship between *T*_*c*_ and *Q* when *ρ*_0_ = 0.0228 Ω⋅m) and is not affected by *ρ*_0_. The value of *T*_*c*_ showed the NW tip to be far from the 1599 K melting point of CuO. This suggested that melting could not be responsible for initiating the breakdown. The resistivity of the CuO NWs decreased as the temperature increased, thus creating a negative feedback on the Joule heating. Due to the negative feedback, the rise in temperature was limited before the critical point. On the other hand, if the emission current increases above the critical point, the balance between the Joule heating and thermal dissipation will be broken and a thermal runaway will ensue. This is the only mechanism that can initiate breakdown in a CuO NW.

Under *in situ* experimental conditions, it is challenging to maintain the consistency of physical parameters such as the radius and length of the NWs. However, a model of the influence of these parameters is helpful when experimentally investigating the field emission properties. To derive this, we set the values 0.32 eV for *Q*, 0.0228Ω⋅m for *ρ*_0_, and 33 W/(m⋅K) as the heat conduction coefficient.

**Figure 3:**
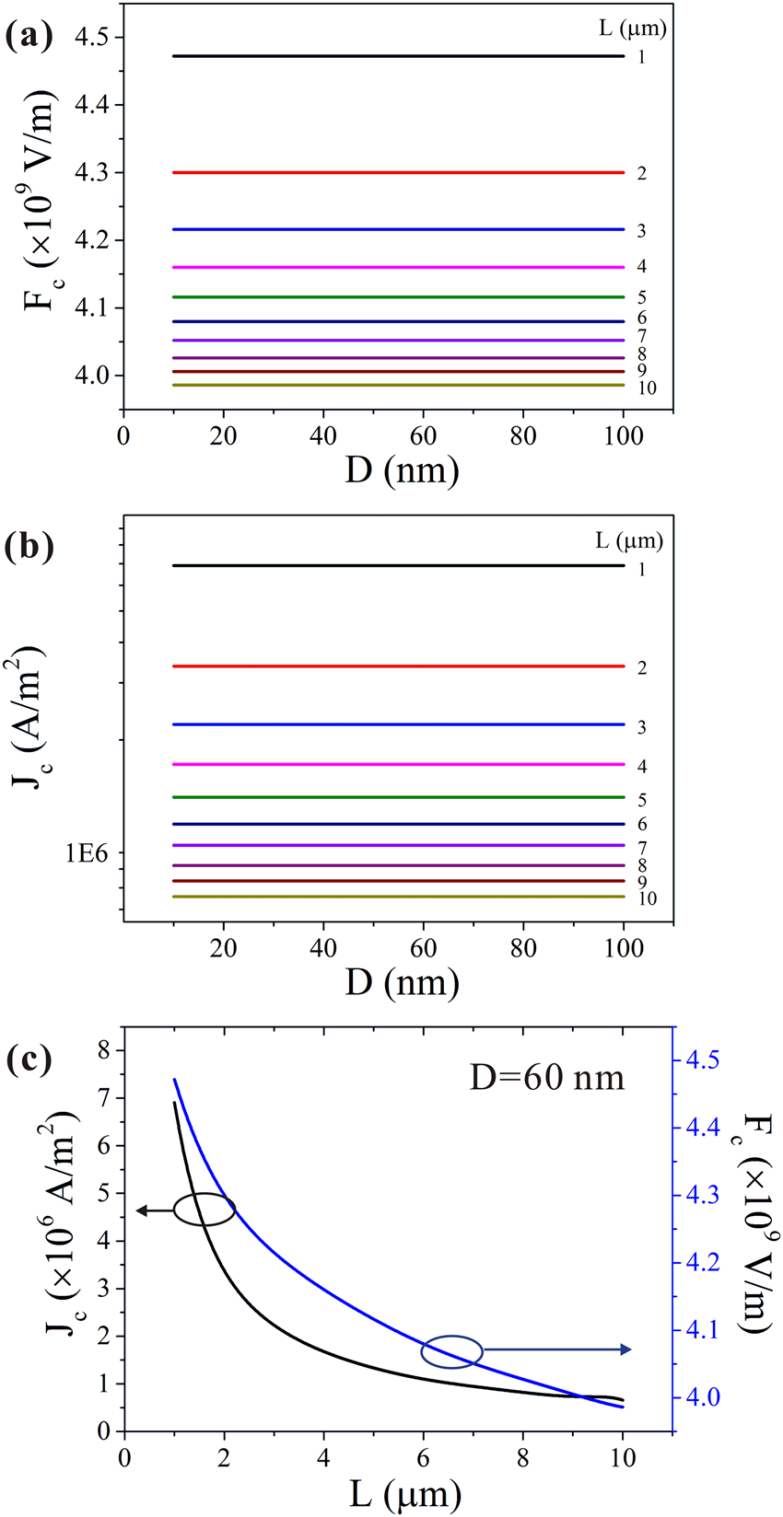
Diameter dependence of the maximum local electric field (**a**) and maximum emission current density (**b**) of CuO NWs with different lengths. (**c**) Relationship between the length and the maximum emission current density and maximum local electric field.

As can be seen from Fig. 3, no change in *J*_*c*_ was observed when the diameter of the NW was varied, but it decreased nonlinearly as the length was increased. When the length of the NW was increased from 1 μm to 10 μm, the change of *J*_*c*_ reached 1 order. The value of *F*_*c*_ was also unrelated to the diameter of the NWs. It should be noted that *F*_*c*_ is the local electric field, rather than the macroscopic applied field. It is well known that the effect of the radius and the length in the macroscopic applied field is reflected by the factor *β* (see Supplementary Fig. S2).

## Experimental results

NWs of different resistivities were used to investigate electric conductance and field emission property. NWs were weld on the tungsten probe. Due to the excellent heat dissipation of the tungsten and its large volume compared with the nanowire, the temperature of the tungsten probe is assumed to be fixed at temperature T_0_.

In the measurement of field emission, an applied voltage was increased step by step from 0 to the target value. In the first round of measurements, the target voltage was set, based on experience, at a level that would prevent damage being caused to the NW, while allowing the field emission phenomenon to be observed. The target voltage was increased gradually in subsequent measurement rounds until NW breakdown was observed from the emission current or by inspection of the scanning electron microscopy (SEM) image.

**Figure 4:**
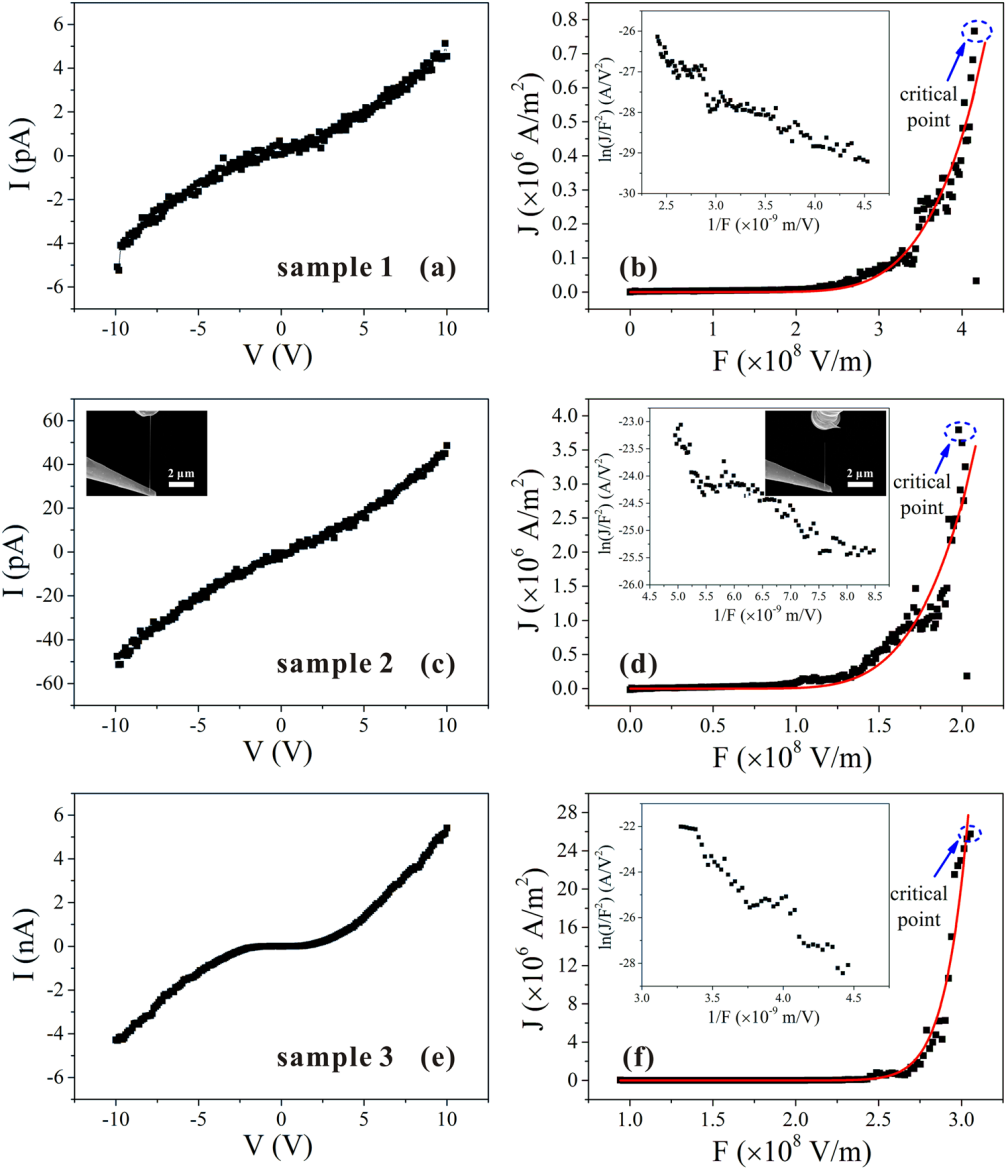
(**a**,**c**,**e**) Electrical *I*–*V*. (**b**,**d**,**f**) Field emission *I*–*V* of individual CuO NWs of different resistivities. Sample 1 showed the highest resistivity and Sample 3 the lowest. Insets in (**b**), (**d**), and (**f**) show the corresponding F–N plots. SEM image taken during electrical measurement is given as an inset in (**c**), and an SEM image of the field emission measurement is given as an inset in the F–N plot of Sample 2.

**Figure 5:**
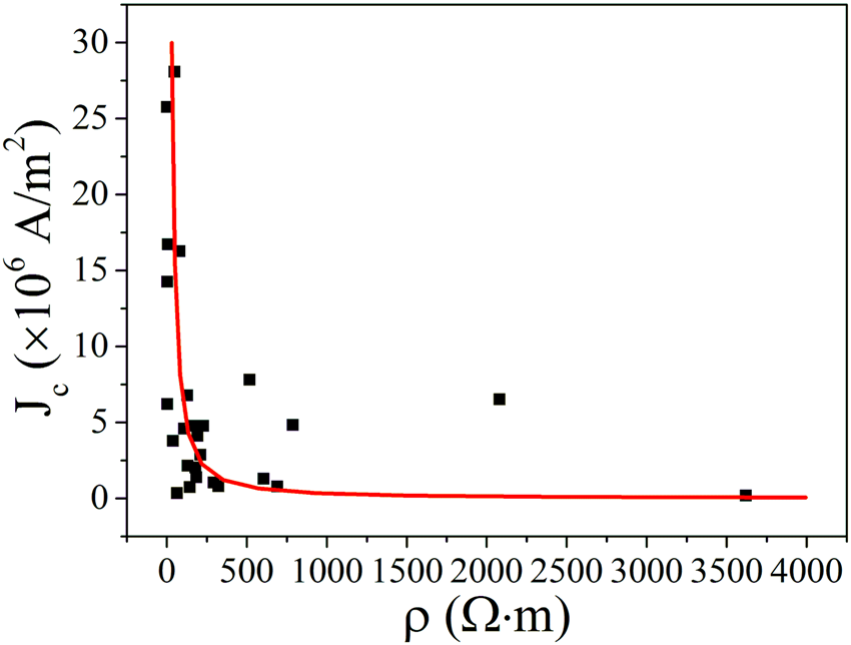
Statistical results for resistivity dependence of *J*_*c*_. Scattered symbols are the experimental results, and the red line shows the simulation results from our semiconductor F–E model at different values of *ρ*_0_ and *Q*.

The results of the last round of field emission measurements for the three samples are shown as Fig. 4(b),(d) and (f). Although experimental data for the low emission current of a p-type CuO NW contain noise, this does not affect the results for the maximum emission current density. The electrical measurement results showed high replicability, so that only one result is shown for each sample in Fig. 4(a),(c) and (e). Table 1 lists the values of the other sample parameters. As can be seen, the maximum field emission current density was higher for NW with lower resistivity. Figure 5 shows the statistical results for all NWs tested, and more specific information on the related parameters is given in Supplementary Fig. S3.

**Table 1:** Summary of measurement results shown in Fig. 4.

No.	1	2	3
Diameter (nm)	38.9	34.5	56.8
Length (μm)	3.1	4.4	2.2
Resistivity (Ω·m)	691	37.8	1.4
Maximum current density (A/m^2^)	7.66 × 10^5^	3.8 × 10^6^	2.57 × 10^7^

The maximum field emission current *J*_*c*_ increased as the resistivity decreased, matching the simulation results. However, the magnitude of change in *J*_*c*_ for nanowires with different resistivity increased more than two orders, which could not be explained by simulation results using a single *Q*. Even if the length of the nanowire varies from 1.46 μm to 8.3 μm (the shortest and longest length of nanowires in the experiment respectively), only an approximately four-fold increase in the value of *J*_*c*_ is produced. A different value of *Q* was therefore needed in the fitting calculation, suggesting that different conduction mechanisms may be present. This was consistent with the results for the temperature dependence of the electrical property (see Supplementary Fig. S4), which showed that the value of *Q* increased with resistivity. We assumed that different values of *Q* were induced by the mix of transport mechanisms, as the values ranged from 200 meV to 500 meV. This could not be accounted for by the existence of such a wide range of defect energy levels.

The fitting calculation was repeated using different values of *ρ*_0_ and *Q*, and the results are shown in Fig. 5. The value of *Q* ranged from 200 to 500 meV, which coincides with the value of *Q* in the results of the study for electrical property. *ρ*_0_ is tuned by *Q* and *ρ* as follows: $\rho ={\rho }_{0}\exp \,(\frac{Q}{kT})$. The value interval of *ρ* was set from the experimental results. As can be seen, a good fit was observed between the experimental results and the predictions of the theory.

## Discussion

In the case of a metal, both the resistivity and the density of the emission current will increase in the course of the emission process, and Joule heating will vary significantly. To achieve thermal equilibrium, heat dissipation must take place through thermal conduction and radiation. However, in the case of semiconductor NWs, the temperature-dependent resistivity obeys a different law, with resistivity decreasing as the temperature increases. The two parameters that determine the Joule heating rate therefore pull in opposite directions, and Joule heating is expected to change less than in the case of a metal. Whether thermal equilibrium can be achieved depends on the variation in Joule heating, which is determined in turn by the variation in the current density and resistivity. However, any decrease in resistivity will promote the transportation of electrons, with a corresponding increase in the number of electrons supplied. This will increase the emission current density. The variation in emission current density is therefore also dependent on the variation in resistivity, and the variation in resistivity with temperature, which is related to *Q*, is the key factor that determines whether dynamic equilibrium can be reached. As *Q* becomes larger, the changes in the emission current density and resistivity become more significant. Joule heating will increase, raising the temperature of the NW. Dynamic equilibrium will be more difficult to achieve and thermal runaway will be readily initiated. The maximum emission current *J*_*c*_, the maximum local electric field *F*_*c*_ and the critical temperature of the apex *T*_*c*_ of the NWs will be lower. Conversely, when *Q* is smaller, *J*_*c*_, *F*_*c*_ and *T*_*c*_ will be higher. When *Q* is larger, the corresponding resistivity of the NW will be higher. These observations further explain the mechanisms underlying the phenomena reported here.

By deriving a semiconductor field emission formula and applying it to the temperature-dependent transportation mechanism, new insights have been gained into the parameters that determine the maximum emission from semiconductor NWs. Our findings demonstrate that the transportation mechanism plays an important role in determining the maximum emission current. The value of this current is not only influenced by the resistance of the semiconductor NW but also by the transportation mechanism. Our results will support the design of semiconducting NW field emitters with high emission current and will promote the development of novel emitters.

In summary, the maximum field emission current density of semiconductor NWs of different electrical conductivities was predicted using a theoretical model, and the results were confirmed by *in situ* experimental measurements. The model took account of the influence of Joule heating on defect-related semiconductor field emissions. Our experimental results suggested the existence of multiple conduction mechanisms in NWs of different conductivities and could be explained well by applying different values of *ρ*_0_ and *Q* in the semiconductor field emission model. The value of *Q* was shown to influence the critical temperature at the apex of the NW and to restrict thermal runaway to be the only breakdown mechanism that applies to CuO NWs. These findings also suggest that both the concentration of defects and the electrical transport mechanisms play important roles in determining the maximum emission current density of a semiconductor NW.

## Methods

### Material preparation

CuO NWs were grown using the thermal oxidation method. Full details of the preparation process, an analysis of the composition of the wires, and a structural characterization were presented in a previous work^38^.

### Measurements

A modified scanning electron microscopy (Zeiss Supra 55) system was used to measure the electrical characteristics and field emission properties of the individual CuO NWs. The system was equipped with two precisely manipulated tungsten probes. Before measurements were conducted, a thin layer of amorphous carbon film was coated onto the contact point between the NW and the probe. This process took about 30 min. After the process, nanowires were welded onto the tungsten probe. Then, the probe was pulled in the direction opposite to the substrate, and the nanowires were separated from the substrate. To measure the electrical properties, a second probe was brought into contact with the apex of the NW. To ensure contact stability, a thin layer of amorphous carbon film was applied to this contact point approximately 5 min. Field emission measurements were carried out after the electrical conduction measurements. The probe was moved back approximately 1 μm. All the *I*–*V* properties were measured using a programmable picoammeter (Keithley 6487). During the measurement process, the pressure in the vacuum chamber was maintained at ~10^−4^ Pa.

## Electronic supplementary material

Supplementary Information
