# Supplementary material for: Maximum field emission current density of CuO nanowires: theoretical study using a defect-related semiconductor field emission model and in situ measurements
Source: Sci Rep. 2018 Feb 1;8:2131. doi: 10.1038/s41598-018-20575-y (PMC5794980; doi:10.1038/s41598-018-20575-y)
Supplement: Supplementary file 1 — Supplementary Information [file 41598_2018_20575_MOESM1_ESM.doc]

**Maximum field emission current density of CuO nanowires: theoretical study using a defect-related semiconductor field emission model and *in situ* measurements**

Zufang Lin, Peng Zhao, Peng Ye, Yicong Chen, Haibo Gan, Juncong She, Shaozhi Deng, Ningsheng Xu, and Jun Chen^*^

State Key Laboratory of Optoelectronic Materials and Technologies, Guangdong Province Key Laboratory of Display Material and Technology, School of Electronics and Information Technology, Sun Yat-sen University, Guangzhou 510275, China

^*^E-mail: [stscjun@mail.sysu.edu.cn](mailto:stscjun@mail.sysu.edu.cn)

**S1. Relationship between** $T_{c}$ **and** $Q$ **when** $\rho_{0}=0.0228 \text{ }\Omega\cdot\text{m.}$

The relationship between $T_{c}$ and $Q$when $\rho_{0}=0.0228 \text{ }\Omega\cdot\text{m}$is calculated by using $E_{c}=-4.07 eV$, $E_{v}=-5.42 eV$, $k=33 W/(m\cdot K)$ *r* = 30 nm, and *L* = 5 μm. The calculated result is shown in FIG. S1.


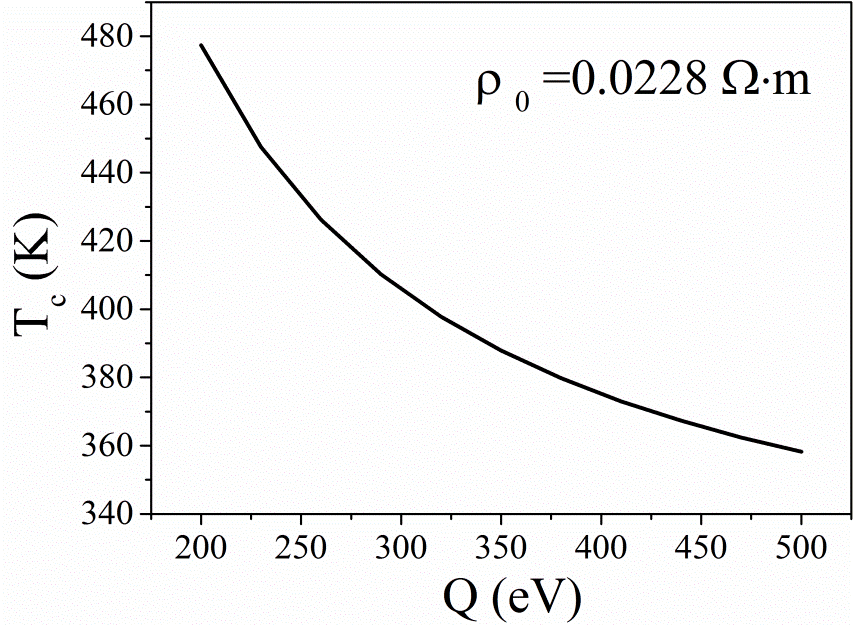


FIG. S1. Relationship between $T_{c}$ and $Q$.

**S2. Diameter dependence of the maximum macroscopic applied field.**

We used the following formula to calculate the enhancement factor $\beta$ of the NWs :

$\beta=1.2(2.5+\frac{h}{r})^{0.9}[1+0.013(\frac{d-h}{d})^{-1}-0.033(\frac{d-h}{d})].$ (1)

The maximum macroscopic applied field was then given by

$F_{macroscopic-c}=F_{c}/\beta.$ (2)

From the FIG. S2 we can see that macroscopic applied field decreases with decreasing diameter and increasing length.


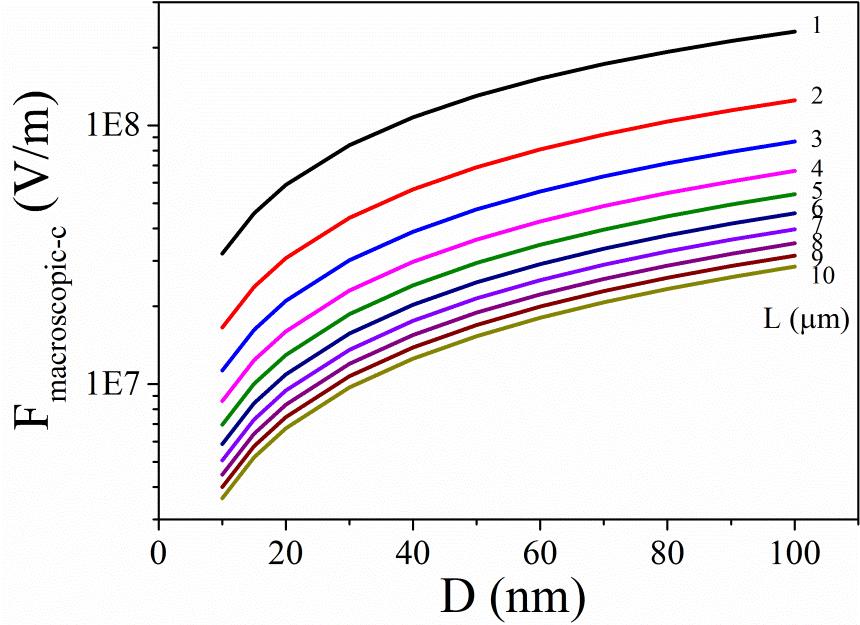


FIG. S2. Diameter dependence of the maximum microscopic applied field of CuO NWs with different lengths.

**S3. Statistical measurement results for individual CuO NWs.**

Table S1: Measured values for 26 individual CuO NWs.

| No. | Diameter (nm) | Length (μm) | Resistivity (Ω·m) | Maximum current density (A/m^2^) |
| --- | --- | --- | --- | --- |
| 1 | 38.9 | 3.06 | 691 | 7.66 × 10^5^ |
| 2 | 34.5 | 4.39 | 37.8 | 3.8 × 10^6^ |
| 3 | 56.8 | 2.19 | 1.4 | 2.57 × 10^7^ |
| 4 | 39 | 5.55 | 517.3475 | 7.79 × 10^6^ |
| 5 | 52.3 | 6.16 | 323.4097 | 8.03 × 10^5^ |
| 6 | 36.5 | 4.21 | 2080.41 | 6.52 × 10^6^ |
| 7 | 57.2 | 2.86 | 192.858 | 4.11 × 10^6^ |
| 8 | 55.8 | 2.79 | 787.7357 | 4.83 × 10^6^ |
| 9 | 49.6 | 2.48 | 226.3311 | 4.77 × 10^6^ |
| 10 | 39 | 2.97 | 79.53305 | 1.63 × 10^7^ |
| 11 | 29 | 2.97 | 178.0337 | 2.02 × 10^6^ |
| 12 | 26.6 | 1.46 | 107.8541 | 4.58 × 10^6^ |
| 13 | 33.3 | 2.33 | 292.922 | 1.05 × 10^6^ |
| 14 | 70 | 6.12 | 144.2664 | 7.29 × 10^5^ |
| 15 | 60 | 2.34 | 8.02278 | 1.67 × 10^7^ |
| 16 | 38 | 1.9 | 604.37 | 1.29 × 10^6^ |
| 17 | 79.5 | 4.7 | 3620.298 | 1.79 × 10^5^ |
| 18 | 109.8 | 3.96 | 63.00477 | 3.39 × 10^5^ |
| 19 | 83.9 | 2.46 | 3.28521 | 6.21 × 10^6^ |
| 20 | 60 | 2.19 | 3.9333 | 1.43 × 10^7^ |
| 21 | 62.1 | 7.94 | 127.204 | 6.78×10^6^ |
| 22 | 40 | 6.12 | 165.6741 | 4.77 × 10^6^ |
| 23 | 30 | 5.04 | 49.05499 | 2.81 × 10^7^ |
| 24 | 88.7 | 8.3 | 210.6775 | 2.86 × 10^6^ |
| 25 | 99.7 | 4.95 | 129.6125 | 2.16 × 10^6^ |
| 26 | 42.3 | 4.72 | 184.6966 | 1.38 × 10^6^ |

**S4. Relationship between** $\rho$ **and** $Q$**obtained from experimental measurements.**

Twenty-eight single CuO NWs were chosen for measurement of the temperature dependence of the *I*–*V* characteristics. Tests were conducted on a Cascade probe station using a semiconductor parameter analyzer (Agilent Technologies B1500A). The two-electrode measurement structures were fabricated using ultraviolet photolithography. A typical SEM image of the structure is shown as an inset at the upper left of FIG. S3(a). The temperature dependence of the *I*–*V* property is shown in FIG. S3(a). The inset at the bottom right of FIG. S3(a) is a typical Arrhenius plot of one sample, which could be fitted well by a straight line. The value of $Q$ could be determined from the Arrhenius plot. $Q$ is a parameter that reflects the transport mechanism, which is influenced in turn by the concentration of defects. $Q$ will take a low value, typically of a few dozen meV for the nearest–neighbor hopping (NNH) mechanism and is related to the defect energy level in the case of thermal activation, which is in the order of a hundred meV. The values of $Q$ for NWs of different resistivities are shown in FIG. S3(b). The value of $Q$ increased as the resistivity increased. Specific values are given in Table S2. We assume that different values of $Q$ were induced by the mix of transport mechanisms, as the values ranged from 200 meV to 500 meV. This could not be accounted for by the existence of such a wide range of defect energy levels.


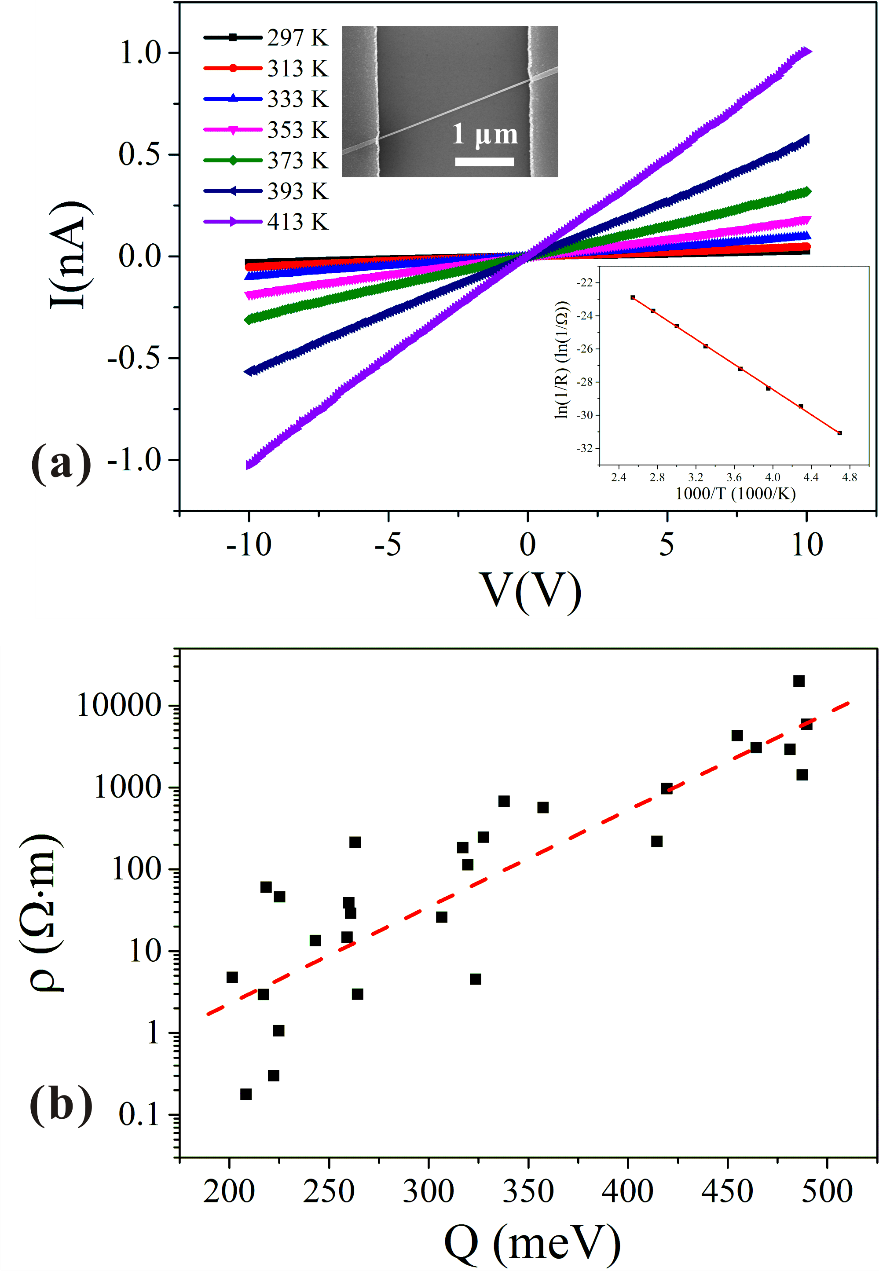


FIG. S3. (a) Temperature dependence of the *I*–*V* properties of the NW. SEM image of the measurement structure is given at the bottom left, while the inset at the upper right shows the logarithm of reciprocal resistivity as a function of reciprocal temperature (Arrhenius plot), for a single CuO NW sample. (b) Measured values of $Q$ for NWs of different resistivities. Scatter plot shows the experimental results.

Table S2: Specific values for 28 individual CuO NWs.

| No. | Resistivity (Ω·m) | $Q$ (meV) |
| --- | --- | --- |
| 1 | 2905.098 | 481.215 |
| 2 | 214.1158 | 263.1962 |
| 3 | 26.05289 | 306.6031 |
| 4 | 13.40606 | 243.1392 |
| 5 | 0.17772 | 208.4426 |
| 6 | 964.2502 | 419.4251 |
| 7 | 4309.291 | 454.8619 |
| 8 | 19938.64 | 485.7318 |
| 9 | 46.06118 | 225.2432 |
| 10 | 245.739 | 327.5376 |
| 11 | 182.9175 | 317.0574 |
| 12 | 0.30075 | 222.2564 |
| 13 | 4.525 | 323.5905 |
| 14 | 2.96485 | 264.2685 |
| 15 | 3071.46978 | 464.315 |
| 16 | 5897.03042 | 489.75651 |
| 17 | 569.9298 | 357.4671 |
| 18 | 2.94025 | 217.205 |
| 19 | 28.84379 | 260.8959 |
| 20 | 14.72283 | 259.1322 |
| 21 | 39.03896 | 260.0224 |
| 22 | 675.9621 | 337.8059 |
| 23 | 4.76585 | 201.493 |
| 24 | 113.3242 | 319.7102 |
| 25 | 60.22848 | 218.4283 |
| 26 | 1.06431 | 224.7962 |
| 27 | 218.767 | 414.5573 |
| 28 | 1424.292 | 487.411 |

**S5. Field emission I-V curves and corresponding SEM images of the nanowires during conditioning process.** In order to reduce the impact of the contaminants and residue molecules on the experimental results, a conditioning process was carried out in the field emission measurement by ramping up and down the applied voltage. And after multiple rounds of ramping voltage, the field emission I-V curves of the nanowires showed high replicability before breakdown as shown in Fig. S4 (a). This result indicates that the field emission current is stable and there is no rapid rise in the emission current. Moreover, we do not notice any changes in geometry of the nanowire in the field emission measurements before breakdown. Fig. S4 (b) shows the SEM images of the morphology of the nanowire taken after each round of the field emission measurement. Therefore, we think that field enhancement change due to the emitter tip geometry sharpening can be neglected before breakdown.


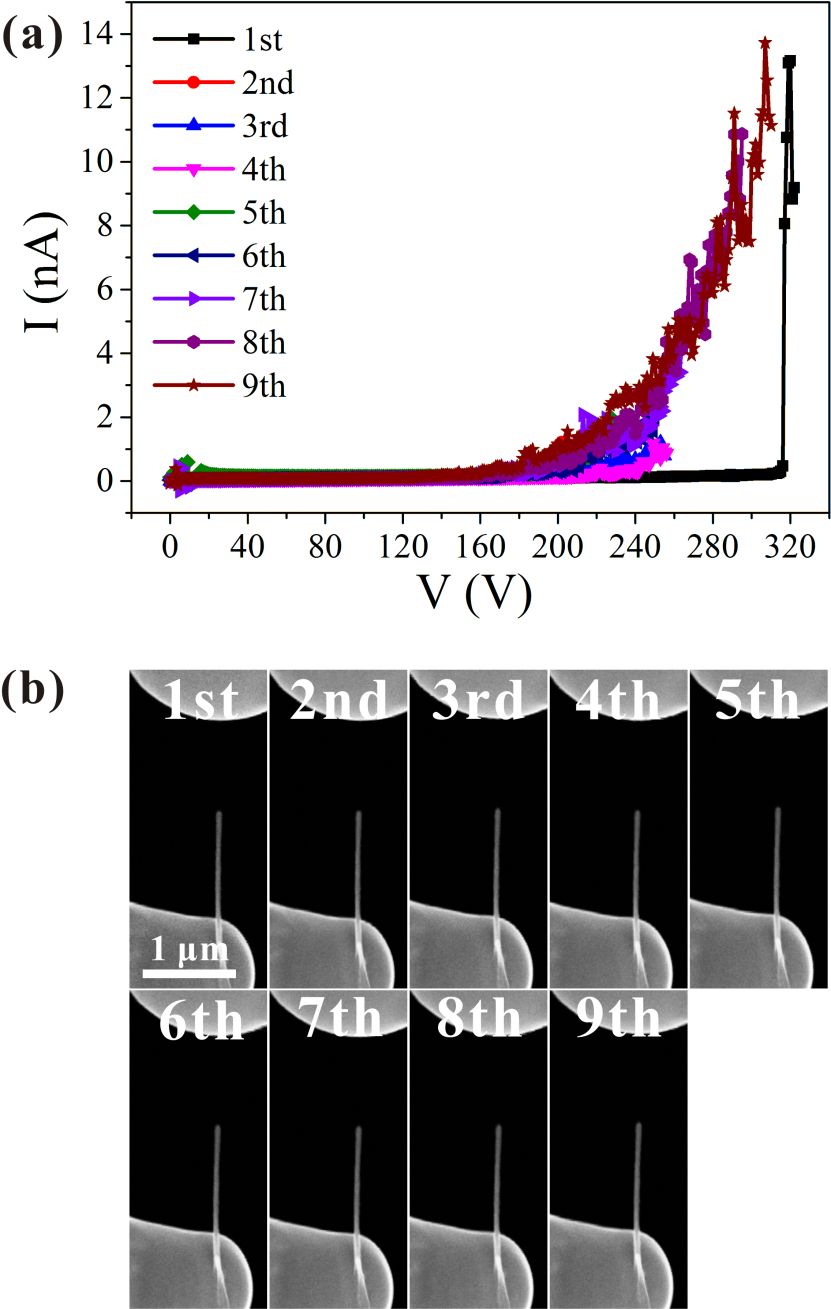


FIG. S4. (a) Field emission I-V curves of the nanowires measured from 9 rounds of ramping applied voltage. (b) Corresponding SEM images taken after each round of the field emission measurement. Magnification is same for all the images.

**References**

1 Bonard, J. M., Dean, K. A., Coll, B. F. & Klinke, C. Field emission of individual carbon nanotubes in the scanning electron microscope*. Phys. Rev. Let*t**.** 89, 197602 (2002).
